# Expressive Attribute-Based Proxy Signature Scheme for UAV Networks

**DOI:** 10.3390/s26010055

**Published:** 2025-12-21

**Authors:** Lei He, Yong Gan, Songhe Jin

**Affiliations:** School of Computer Science and Technology, Zhengzhou University of Light Industry, Zhengzhou 450000, China

**Keywords:** expressive, attribute-based signature, proxy signature, unmanned aerial vehicle network

## Abstract

Unmanned aerial vehicle (UAV) networks have become an essential component of modern civilian and military infrastructures. However, the communication channels between UAVs and their control entities remain vulnerable to spoofing and message tampering attacks. Although conventional digital signature schemes can ensure message authentication and integrity, they often undermine the real-time responsiveness of UAV operations and fail to protect the privacy of signers. To address these limitations, we propose an expressive attribute-based proxy signature (EABPS) scheme tailored for UAV networks. The scheme enables fine-grained authorization and authentication, ensuring that only entities whose attributes satisfy a specified access structure can generate valid proxy signatures. Furthermore, the scheme preserves signer privacy by decoupling signatures from explicit identities. Comprehensive security analysis and extensive experimental evaluation demonstrate that the proposed EABPS scheme achieves strong security guarantees while offering improved computational efficiency and expressiveness, making it a practical solution for secure communication in UAV networks.

## 1. Introduction

**Figure 1:**
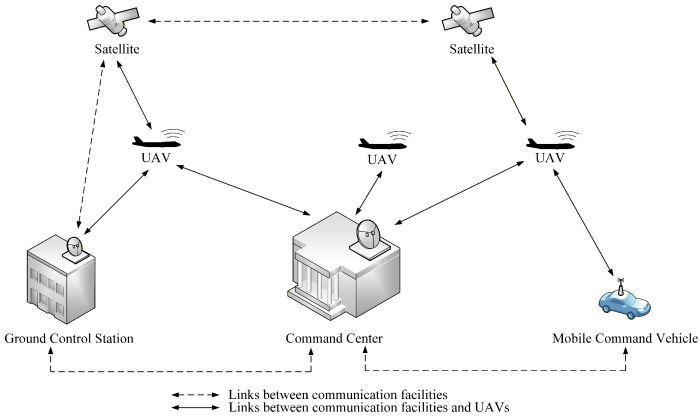
UAV network.

Unmanned aerial vehicle (UAV) networks are emerging as a critical infrastructure for a wide range of civilian and military applications. A typical UAV network consists of aerial platforms equipped with sensors and communication modules, ground control stations that coordinate missions, and a command center responsible for making strategic decisions, and sometimes satellites or mobile relays that extend coverage. It is illustrated in Figure 1. Unlike conventional wireless networks, UAV networks are characterized by high mobility, rapidly changing topology, and intermittent connectivity, which result from the dynamic flight patterns of UAVs and the variability in wireless links [1]. These features make UAV networks highly flexible and capable of providing on-demand services in scenarios such as disaster response, environmental monitoring, and surveillance. However, the same characteristics also introduce unique challenges for secure and reliable communication. The limited energy, computation, and bandwidth resources of UAVs further complicate the design of security schemes.

Despite these advantages, UAV networks face significant security threats due to their exposure to open and often hostile environments. Communication links between UAVs and control entities are vulnerable to eavesdropping, spoofing, and message tampering, which may lead to mission failure or unauthorized control of UAVs. Ensuring the integrity and authentication of transmitted commands is therefore a critical requirement, which can be achieved through the implementation of a digital signature scheme. Conventional digital signature schemes provide a foundation for securing these communications; however, they often rely on digital certificates or identity-based signatures, which may not be suitable for dynamic and heterogeneous UAV environments. In practice, it is sometimes necessary to delegate signing authority to intermediate nodes, such as ground control stations, to reduce communication delays and maintain operational continuity. Moreover, in scenarios where privacy preservation is essential, digital certificates and identity-based signatures may inadvertently reveal sensitive information about the command issuer. These limitations motivate the exploration of proxy signature schemes and attribute-based signature (ABS) schemes, which can jointly provide flexible delegation, fine-grained access control, and signer’s privacy.

To address these challenges, we propose an expressive attribute-based proxy signature (EABPS) scheme specifically designed for UAV networks. Unlike traditional schemes that either focus solely on delegation or on attribute-based access control, our approach integrates both in a unified framework while enhancing expressiveness. In particular, the proposed scheme supports complex attribute policies that enable fine-grained authorization, ensuring that only entities whose attributes satisfy predefined conditions can act as proxy signers. This capability not only strengthens security but also accommodates the dynamic and heterogeneous nature of UAV networks. Furthermore, the scheme preserves signer anonymity by decoupling signatures from explicit identities, thereby mitigating privacy risks in sensitive applications. By balancing strong security guarantees with computational efficiency suitable for resource-constrained UAVs, the proposed scheme offers a practical solution for secure communications in UAV networks.

### 1.1. Problem Statement

In a UAV network, the command center usually holds the highest authority and is responsible for issuing instructions that guide UAVs to perform diverse tasks. However, direct communication between the command center and UAVs often faces practical limitations. Due to the wide deployment range of UAVs, communication links may be long-distance, intermittent, or subject to poor channel quality, making them highly vulnerable to interception and modification by adversaries. Although the command center and other infrastructure components may possess sufficient computational power and stable communication resources, UAVs themselves are constrained by limited energy, processing capacity, and memory, which amplifies the impact of insecure or delayed transmissions.

To ensure that UAVs execute only authentic commands, a digital signature scheme is indispensable. However, conventional digital signature schemes introduce two major challenges. First, the inherent delay in transmitting signed commands from a remote command center to UAVs can degrade real-time responsiveness, which is crucial in time-sensitive missions. Second, traditional signature schemes that use digital certificates and identity-based signature schemes couple authentication with user identity, exposing sensitive information about the signer and creating potential privacy risks.

In many deployment scenarios, it is desirable to temporarily delegate signing authority to nearby entities, such as ground control stations or mobile command units, so that UAVs can receive authenticated commands with reduced latency. At the same time, privacy-sensitive missions require that UAVs verify the authenticity of commands without learning the identity of the signer. These requirements highlight the necessity for a signature scheme that simultaneously supports secure delegation and privacy preservation. An attribute-based proxy signature (ABPS) scheme naturally addresses these needs by allowing for flexible delegation of signing rights while decoupling signatures from explicit identities.

### 1.2. Our Contributions

Motivated by the limitations of existing approaches, this paper introduces an ABPS scheme designed for UAV networks. Unlike prior works that mainly rely on threshold predicates, our scheme adopts a tree-based access structure, thereby providing greater expressiveness and flexibility. The key contributions of this work are summarized as follows:

- Expressive access control: In contrast to the previous works, our scheme employs a tree-based access structure that provides higher expressiveness than traditional threshold policies. This enhancement allows the delegation and signing policies to express multi-branch, hierarchical, and fine-grained authorization and authentication conditions that can not be represented by threshold structures. The use of a tree-based access structure not only increases the flexibility of authorization and authentication but also makes the scheme better suited for UAV network environments.
- Integration of proxy signature schemes and ABS schemes: Our scheme unifies the advantages of proxy signature schemes and attribute-based signature schemes, allowing a command center to securely delegate signing authority to a proxy entity while ensuring that only proxies with the required attributes can generate valid signatures.
- Privacy preservation: By binding signatures to attributes rather than explicit identities, our scheme ensures that the identities of both the original signer and the proxy signer remains hidden. This property is particularly important in sensitive UAV applications where anonymity of command issuers must be preserved.

In summary, the proposed scheme advances the state of the art by enabling expressive, privacy-preserving, and delegation of signing rights in UAV networks.

### 1.3. Organization of This Paper

The remainder of this paper is organized as follows: Section 2 reviews related work on security solutions for UAV networks and ABS schemes. Section 3 introduces the necessary preliminaries. Section 4 presents the formal model and security model of the proposed EABPS scheme. Section 5 details the design of the EABPS scheme for UAV networks. Section 6 provides a comprehensive analysis of the scheme, covering correctness, security, and efficiency. Finally, Section 7 concludes this paper and discusses possible directions for future research.

## 2. Related Work

### 2.1. Security Solutions for UAV Networks

Due to the open and dynamic nature of UAV networks, their communication links are highly vulnerable to a variety of attacks. Zhi et al. analyzed the security and privacy issues of UAVs and emphasized that the communication channels between UAVs and ground control stations are susceptible to attacks [2]. Samanth et al. highlighted the necessity of designing more appropriate digital signature schemes specifically tailored for UAV networks [3].

Khan et al. proposed an identity-based generalized signcryption scheme for flying ad hoc networks [4], but Din et al. demonstrated that this scheme is insecure and presented an improved scheme [5]. Wang et al. proposed an aggregate authentication scheme based on ID-based encryption for UAV cluster networks [6]. Lei et al. proposed an optimized lightweight identity security authentication protocol [7].

Various certificateless signature schemes have been explored in the UAV context. He et al. proposed a certificateless designated verifier proxy signature scheme [8], but Xu et al. later showed its vulnerability to impersonation attacks and suggested an efficient construction [9]. Qu and Zeng presented a certificateless proxy signcryption scheme in the standard model [10]. Sysoyev et al. proposed a certificateless group signature scheme for UAV communications in resource-constrained environments [11].

Li et al. proposed an online/offline revocable identity-based group signature scheme for UAVs [12]. To further enhance lightweight authentication, Liu et al. proposed a trustworthy message exchange scheme combining cryptography with trust management technologies [13]. Aissaoui et al. implemented post-quantum digital signature standards for UAVs and evaluated their performance [14]. Li et al. introduced a secure and reliable UAV network service architecture and a UAV cluster identity management module [15]. Khan et al. proposed a certificate-based proxy signature scheme for UAV communications, which uses hyperelliptic curve cryptography [16].

A comparison of representative security solutions is summarized in Table 1.

The comparison in Table 1 shows that existing security solutions for UAV networks generally provide integrity and authentication, which are fundamental requirements for secure communication. However, these schemes can not protect the identity of the signer. This means that sensitive information about the signer may be exposed, which is unacceptable in scenarios that require anonymity or privacy protection. Therefore, although these schemes are effective for basic security services, they do not meet the requirement of identity protection in UAV environments.

### 2.2. Attribute-Based Signature Schemes

ABS has been widely investigated as a promising primitive for providing fine-grained authentication and privacy protection. Maji et al. first formalized ABS and presented a concrete construction in the generic group model [17]. Since then, many extensions have been proposed. Su et al. proposed expressive attribute-based signature scheme [18]. This work introduced the idea of using expressive access structures to control signing capabilities, and it supports signer privacy and unforgeability. Liu et al. constructed a revocable and comparable lattice-based ABS scheme from lattices [19]. In response to the security risks posed by key leakage, Wu et al. proposed a puncturable ABS scheme [20].

ABS schemes with a single attribute authority suffer from centralization issues. To address this issue, Li et al. proposed a multi-authority ABS scheme [21]. Su et al. proposed a distributed attribute-based signature scheme that contains multiple attribute authorities [22].

Outsourcing has been used to reduce computational costs. Chen et al. presented two outsourced ABS schemes [23]. Tao et al. proposed a lightweight decentralized ABS scheme, which outsources complex computation of the signer to a cloud server [24]. Xiong et al. developed a server-aided ABS scheme with expressive access structure [25]. Huang and Lin proposed a server-aided ABS scheme with perfect anonymity [26]. Li et al. proposed a decentralized server-aided ABS scheme in the Internet of things [27].

Beyond ABS, the ABPS paradigm has been introduced to enable delegation with fine-grained attribute-based control. Liu et al. defined the ABPS model and proposed an initial construction [28]. He et al. subsequently designed ABPS schemes for UAV networks, demonstrating their feasibility and security [29], and further extended them with flexible threshold predicates [30]. He et al. also proposed an efficient threshold ABPS scheme for UAV networks [31]. While these works represent important progress, existing ABPS schemes are still largely constrained to threshold-based access structures, which limits their ability to capture complex and dynamic authorization requirements in the UAV environments. It is important to design an expressive ABPS scheme that leverages tree-based access structures in order to achieve fine-grained, flexible, and privacy-preserving signature suitable for UAV networks.

A comparison of representative ABPS schemes is summarized in Table 2.

Table 2 shows that existing ABPS schemes already provide integrity, authentication, and protection of the signer’s identity. However, these schemes adopt threshold-based access structures, which limit their expressiveness and prevent support for more fine-grained authorization and authentication policies. Our EABPS adopts a tree-based access structure, which allows for more flexible and expressive delegation and signing policies and is better suited for complex UAV application scenarios.

In summary, the existing security solutions for UAV networks mainly focus on ensuring integrity and authentication but lack protection of signer’s identity. Traditional ABPS schemes already provide identity protection, yet they rely on threshold-based access structures that limit their flexibility. These structures cannot represent complex or hierarchical authorization and authentication requirements that often arise in UAV applications. Our EABPS scheme addresses these limitations by adopting a tree-based access structure with higher expressiveness. This enables fine-grained control of signing rights while preserving signer identity privacy, thus providing a more powerful and suitable solution for UAV environments.

## 3. Preliminaries

### 3.1. Complexity Assumption

**Definition 1** (Computational Diffie-Hellman (CDH) Problem)**.** Let G be a cyclic group of prime order q with generator g. For unknown integers $x,y\in Z_{q}^{*}$, the CDH problem is to compute $g^{xy}$ given the tuple $\left(g,g^{x},g^{y}\right)$.

### 3.2. Lagrange Interpolation

Let *S* be a set of *d* distinct points in the finite field $Z_{q}$. Any polynomial t(x) of degree at most d−1 over $Z_{q}$ is uniquely determined by its values {t(j)∣j∈S}. Specifically, for any point $i\in Z_{q}$, the value t(i) can be reconstructed using the Lagrange interpolation formula $t\left(i\right)=\sum_{j\in S}t\left(j\right)\cdot {∆}_{j,S}\left(i\right)$ where Lagrange interpolation coefficient ${∆}_{j,S}\left(i\right)=\prod_{\eta \in S,\eta \neq j}\frac{i-\eta }{j-\eta }$.

## 4. Formal Model and Security Model

### 4.1. Formal Model

Our EABPS scheme involves four distinct entities within the UAV network architecture: an attribute authority (AA), an original signer (OS), a proxy signer (PS), and a verifier. It is assumed that the OS and PS satisfy the same access structure.

- AA: This fully trusted entity is responsible for initializing the public parameters and generating private keys for OS and PS based on their respective attribute sets.
- OS: This entity, such as a command center, holds the primary authority. The OS can delegate its signing rights to another entity for a specific purpose, which is defined in a warrant. The OS generates a valid delegation only if its attributes satisfy the access structure.
- PS: This entity, for instance, a ground control station, is authorized by the OS to sign messages on its behalf. The PS generates a valid proxy signature only if its attributes satisfy the access structure.
- Verifier: This entity, typically a UAV, validates a received proxy signature to confirm the authentication and integrity of a command.

The formal model of the proposed EABPS scheme consists of seven algorithms: Algorithms 1–7. Each algorithm is responsible for a specific phase in the system. To improve clarity, each algorithm is presented in a graphical step-based format.
**Algorithm 1:** *Setup*$\left(1^{\lambda }\right)$
 **Input:** Security parameter $1^{\lambda }$;
 Generate public parameters params and master secret key MSK;
 **Output:** (params,MSK);

**Algorithm 2:** *KGPri*(params,MSK,ω)
 **Input:** Public parameters, master secret key, attribute set ω;
 Generate private key $sk_{\omega }$ for user with attribute set ω;
 **Output:** $sk_{\omega }$;

**Algorithm 3:** *KGPub*(params,MSK,Γ)
 **Input:** Public parameters, master secret key, attribute tree Γ; Derive corresponding public information based on Γ; **Output:** $pk_{\Gamma }$;

**Algorithm 4:** *DelGen*$\left(params,sk_{\omega_{o}},w\right)$
 **Input:** Public parameters, original signer’s private key $sk_{\omega_{o}}$, warrant *w*; Generate delegation based on $\left(sk_{\omega_{o}},w\right)$; **Output:** Delegation $\sigma_{o}$;

**Algorithm 5:** *DelVer*$\left(params,w,\sigma_{o},pk_{\Gamma }\right)$
 **Input:** Public parameters, warrant *w*, delegation $\sigma_{o}$, public key $pk_{\Gamma }$; Verify the correctness and validity of delegation $\sigma_{o}$; **Output:** Accept or Reject;

**Algorithm 6:** *Sign*$\left(params,sk_{\omega_{p}},\sigma_{o},w,m\right)$
 **Input:** Public parameters, proxy signer’s private key $sk_{\omega_{p}}$, delegation $\sigma_{o}$, warrant *w*, message *m*; Generate proxy signature with the authorized signing capability; **Output:** Proxy signature $\sigma_{p}$;

**Algorithm 7:** *Verify*$\left(params,w,m,\sigma_{p},pk_{\Gamma }\right)$
 **Input:** Public parameters, warrant *w*, message *m*, proxy signature $\sigma_{p}$, public key $pk_{\Gamma }$; Verify correctness of the signature;
 **Output:** Accept or Reject;


### 4.2. Security Model

The security of our proposed EABPS scheme is analyzed based on the standard notion of existential unforgeability. We formalize this property through a security game played between a challenger C and a probabilistic polynomial time (PPT) adversary A. Following the security models for proxy signatures, we mainly consider the two types of adversaries, $A_{\mathrm{II}}$ and $A_{\mathrm{III}}$, which reflect realistic threats in a proxy signature scheme [32].

To make the threat model more explicit, we further describe the capabilities of both adversaries. Both $A_{\mathrm{II}}$ and $A_{\mathrm{III}}$ are modeled as PPT adversaries and are allowed to access all public parameters of the system. They may intercept, replay, or modify transmitted messages in the communication channel. They are also allowed to query some oracles.

The $A_{\mathrm{II}}$ adversary represents a malicious proxy signer or an external attacker who has compromised the proxy signer’s private key ($sk_{p}$). However, the original signer’s private key ($sk_{o}$) remains secure. Therefore, $A_{\mathrm{II}}$ can not generate a valid delegation without access to $sk_{o}$. This adversary’s goal is to forge a valid proxy signature for which it was not legitimately authorized by the original signer.

The $A_{\mathrm{III}}$ adversary represents a malicious original signer or an external attacker who has compromised the $sk_{o}$. In this scenario, the $sk_{p}$ is considered secure. Therefore, $A_{\mathrm{III}}$ can not generate signatures that require $sk_{p}$. This adversary can attempt to misuse its knowledge of $sk_{o}$ to generate unauthorized delegation, but it remains incapable of producing a valid proxy signature that appears to originate from an honest proxy signer without the corresponding $sk_{p}$.

These clarifications ensure that both adversaries are modeled with realistic abilities. Each adversary has partial key exposure, full access to public system components, and full control over the communication channel.

#### 4.2.1. Existential Unforgeability

We define the security notion of existential unforgeability under a selective-policy and chosen-message attack (EUF-SP-CMA). The security is characterized by a game between the challenger C and an adversary A (who can be either $A_{\mathrm{II}}$ or $A_{\mathrm{III}}$).

The game proceeds as follows for a given adversary type:

**Initialization:**  The adversary A first selects and declares a challenge policy $\Gamma^{*}$ that it intends to target for the forgery.
**Setup:**  The challenger C runs the *Setup* algorithm to generate the public parameters params and the master secret key MSK. Depending on the adversary type:
  - If A is $A_{\mathrm{II}}$, C generates the key pairs for the OS and PS. It provides A with params and the proxy signer’s private key, $sk_{p}$. The OS’s private key $sk_{o}$ is kept secret.
  - If A is $A_{\mathrm{III}}$, C similarly generates the key pairs. It provides A with params and the original signer’s private key, $sk_{o}$. The PS’s private key $sk_{p}$ is kept secret.
**Queries:**  The adversary A is granted access to a set of oracles. It can make a polynomially bounded number of queries to $O_{H_{1}}$, $O_{H_{2}}$, $O_{H_{3}}$. It can also make a polynomially bounded number of queries:
  - Private Key Generation Oracle ($O_{KGPri}$): A can request the private key for any attribute set ω.
  - Delegation Generation Oracle ($O_{DG}$): (Only accessible to $A_{\mathrm{II}}$) A can request a delegation on a warrant *w* and a policy Γ.
  - Signing Oracle ($O_{Sign}$): A can request a valid proxy signature on message *m* with warrant *w* and policy Γ.
**Forgery:**  Finally, A outputs a tuple $\left(w^{*},m^{*},\sigma^{*}\right)$ corresponding to the challenge policy $\Gamma^{*}$.

The adversary $A_{\mathrm{II}}$ wins the game if the forgery meets the following conditions:

1. $A_{\mathrm{II}}$ did not query any $\omega^{*}$ satisfying $\Gamma^{*}\left(\omega^{*}\right)=1$ to $O_{KGPri}$.
2. $A_{\mathrm{II}}$ did not query the pair ($w^{*}$, $\Gamma^{*}$) to $O_{DG}$.
3. $A_{\mathrm{II}}$ did not query the tuple ($m^{*}$, $w^{*}$, $\Gamma^{*}$) to $O_{Sign}$.
4. The signature $\sigma^{*}$ is valid.

The adversary $A_{\mathrm{III}}$ wins the game if the forgery meets the following conditions:

1. $A_{\mathrm{III}}$ did not query any $\omega^{*}$ satisfying $\Gamma^{*}\left(\omega^{*}\right)=1$ to $O_{KGPri}$.
2. $A_{\mathrm{III}}$ did not query the tuple ($m^{*}$, $w^{*}$, $\Gamma^{*}$) to $O_{Sign}$.
3. The signature $\sigma^{*}$ is valid.

**Definition 2** (EUF-SP-CMA)**.** *An ABPS scheme provides EUF-SP-CMA if no PPT adversary $A_{\mathrm{II}}$ and $A_{\mathrm{III}}$ can win the aforementioned game with a non-negligible probability.*

#### 4.2.2. Attribute Privacy for Signers

The EABPS scheme involves two types of signers: the original signer, who generates a delegation on a warrant *w*, and the proxy signer, who generates the final proxy signature on a message–warrant pair (m,w). We define the attribute privacy for an original signer (APOS) by a game between the challenger C and a PPT adversary A.

Since a delegation functions as the original signer’s signature on *w*, we should protect the attribute privacy of the original signer. The goal is to guarantee that a delegation reveals no information about the original signer’s attribute set beyond the fact that it satisfies the access structure Γ. The game is defined as follows:

C runs *Setup*($1^{\lambda }$) and gives params to A. A may query $O_{KGPri}$ and $O_{DG}$.

A chooses two attribute sets $\omega_{0},\omega_{1}$ and a warrant *w* such that $\Gamma \left(\omega_{0}\right)=\Gamma \left(\omega_{1}\right)=1$. C randomly chooses b∈{0,1} and computes $\sigma_{o}$ by using *DelGen* and sends $\sigma_{o}$ to A. Finally A outputs a guess $b^{\prime }$ and wins if $b^{\prime }=b$.

The advantage of A is defined by ${\mathrm{Adv}}_{A}^{APOS}=\left|\mathrm{Pr}\left[b^{\prime }=b\right]-\frac{1}{2}\right|$.

**Definition 3** (APOS)**.** *The EABPS scheme provides attribute privacy for an original signer if ${\mathrm{Adv}}_{A}^{APOS}=\left|\mathrm{Pr}\left[b^{\prime }=b\right]-\frac{1}{2}\right|$ is negligible.*

Similarly, we define the attribute privacy for a proxy signer (APPS) by a game between the challenger C and a PPT adversary A. The goal is to guarantee that a signature reveals no information about the proxy signer’s attribute set beyond the fact that it satisfies the access structure Γ. The game is defined as follows:

C runs *Setup*($1^{\lambda }$) and gives params to A. A may query $O_{KGPri}$, $O_{DG}$ and $O_{Sign}$.

A chooses two attribute sets $\omega_{0},\omega_{1}$ and a message *m* such that $\Gamma \left(\omega_{0}\right)=\Gamma \left(\omega_{1}\right)=1$. C randomly chooses b∈{0,1} and computes $\sigma_{p}$ by using *Sign* and sends $\sigma_{p}$ to A. Finally A outputs a guess $b^{\prime }$ and wins if $b^{\prime }=b$.

The advantage of A is defined by ${\mathrm{Adv}}_{A}^{APOS}=\left|\mathrm{Pr}\left[b^{\prime }=b\right]-\frac{1}{2}\right|$.

**Definition 4** (APPS)**.** *The EABPS scheme provides attribute privacy for a proxy signer if ${\mathrm{Adv}}_{A}^{APPS}=\left|\mathrm{Pr}\left[b^{\prime }=b\right]-\frac{1}{2}\right|$ is negligible.*

The EABPS scheme achieves attribute privacy for signers (APS) if it satisfies both APOS and APPS. This ensures that neither the delegation nor the proxy signature leaks the attribute set used by the signer.

## 5. EABPS Scheme for UAV Networks

### 5.1. Overview of the EABPS Scheme

**Figure 2:**
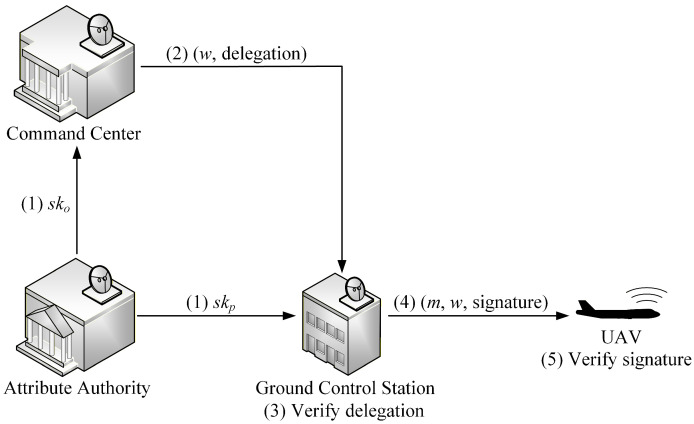
Overall EABPS scheme.

In the proposed EABPS scheme, there are four main entities: an AA, an OS, a PS, and a verifier. The AA is responsible for system initialization and key distribution. The command center acts as the OS and holds an attribute set $\omega_{o}$ together with the corresponding private key $sk_{o}$. The ground control station functions as the PS, equipped with its own attribute set $\omega_{p}$ and private key $sk_{p}$. Finally, the UAV plays the role of the verifier, receiving commands and verifying their integrity and authentication. Unlike threshold-based constructions, the proposed EABPS scheme employs a tree-based access structure Γ, which provides enhanced expressiveness. Both the OS and PS are associated with attributes that must satisfy Γ in order to generate valid signatures. The overall process of our EABPS scheme is illustrated as Figure 2 and can be summarized in the following phases:

(1) The AA executes the *Setup* algorithm to generate public parameters and the master secret key. It then issues private keys corresponding to the attribute sets of the command center and ground control station.

(2) The command center, acting as the OS, generates a warrant *w* and uses its private key $sk_{o}$ to produce a delegation bound to the access structure Γ. The delegation is then securely transmitted to the ground control station.

(3) The ground control station, acting as the PS, verifies the validity of the received delegation. Only if the delegation is correct does the PS proceed.

In our EABPS scheme, the delegation of signing rights is achieved through a warrant and a delegation, rather than the transfer of any secret key. This design ensures that the original signer’s private key is never exposed during the delegation process.

Specifically, the original signer uses its private key to compute a delegation $\sigma_{o}$ that is cryptographically bound to a warrant *w*, which specifies the delegated permissions in a fine-grained manner. The delegation is generated using the *DelGen* algorithm, and it contains no information that allows the proxy signer to recover the original signer’s private key.

The delegation $\sigma_{o}$ is transmitted to the proxy signer. Since $\sigma_{o}$ cannot be used independently to produce signatures and functions only when combined with the proxy signer’s own private key in the *Sign* algorithm, the delegation mechanism prevents misuse and ensures that the signing capability strictly follows the limitations defined in the warrant.

Therefore, the delegation process securely transfers signing rights without transferring any secret signing key.

(4) The PS signs a command message *m* by combining its private key $sk_{p}$ with the delegation. The resulting proxy signature is associated with the attributes satisfying Γ, ensuring that only authorized proxies can produce valid signatures.

(5) The UAV, as the verifier, checks the validity of the proxy signature. If the verification succeeds, the UAV accepts the command as authentic.

Through these phases, the EABPS scheme enables secure delegation and proxy signing in UAV networks.

### 5.2. EABPS Scheme

In this subsection, we present the construction of the EABPS scheme. The proposed EABPS scheme is composed of the following algorithms.

#### 5.2.1. *Setup*

Given the security parameter λ, the algorithm generates two cyclic groups $\left(G_{1},G_{2}\right)$ of prime order *q* with generator $g\in G_{1}$. A bilinear map $e:G_{1}\times G_{1}\to G_{2}$ is defined. The authority selects a random $\alpha \in Z_{q}$, sets $g_{1}=g^{\alpha }$, and chooses a random $g_{2}\in G_{1}$. It then computes $Z=e(g_{1},g_{2})$. Three hash functions are defined: $H_{1}:{\left\{0,1\right\}}^{*}\to G_{1}$ for attributes, $H_{2}:{\left\{0,1\right\}}^{*}\to G_{1}$ for warrants, and $H_{3}:{\left\{0,1\right\}}^{*}\to G_{1}$ for messages. The public parameters are $params=(q,G_{1},G_{2},e,g,g_{1},g_{2},Z,H_{1},H_{2},H_{3})$, and the master secret key is MSK=α.

#### 5.2.2. *KGPri*

The attribute authority generates private key $sk_{\omega_{o}}$ for the original signer with an attribute set $\omega_{o}$. Given the master secret key α and an attribute set $\omega_{o}$, the authority chooses $r_{o}\in Z_{q}$ at random and computes $d_{o}=g_{2}^{r_{o}+\alpha }$. For each attribute $i_{o}\in \omega_{o}$, it selects $r_{i_{o}}\in Z_{q}$ and computes

$$d_{i_{o}0}=g_{2}^{r_{o}/\alpha }\cdot H_{1}{\left(i_{o}\right)}^{r_{i_{o}}},\;d_{i_{o}1}=g^{r_{i_{o}}}.$$

The private key for attribute set $\omega_{o}$ is $sk_{\omega_{o}}=\left(d_{o},{\left\{\left(d_{i_{o}0},d_{i_{o}1}\right)\right\}}_{i_{o}\in \omega_{o}}\right)$.

In the same way, the attribute authority generates private key $sk_{\omega_{p}}$ for the proxy signer with an attribute set $\omega_{p}$. The authority chooses $r_{p}\in Z_{q}$ at random and computes $d_{p}=g_{2}^{r_{p}+\alpha }$. For each attribute $i_{p}\in \omega_{p}$, it selects $r_{i_{p}}\in Z_{q}$ and computes

$$d_{i_{p}0}=g_{2}^{r_{p}/\alpha }\cdot H_{1}{\left(i_{p}\right)}^{r_{i_{p}}},\;d_{i_{p}1}=g^{r_{i_{p}}}.$$

The private key for attribute set $\omega_{p}$ is $sk_{\omega_{p}}=\left(d_{p},{\left\{\left(d_{i_{p}0},d_{i_{p}1}\right)\right\}}_{i_{p}\in \omega_{p}}\right)$.

#### 5.2.3. *KGPub*

For an access tree Γ, the root node is assigned a polynomial $p_{root}$ with $p_{root}\left(0\right)=\alpha$. For each internal node *x* with threshold $k_{x}$, a random polynomial $p_{x}$ of degree $k_{x}-1$ is generated such that $p_{x}\left(0\right)=p_{parent(x)}\left(\mathrm{index}\left(x\right)\right)$. For each leaf node *x* associated with attribute i=att(x), the authority computes $D_{i}=g^{p_{x}\left(0\right)},\;h_{i}=H_{1}{\left(i\right)}^{p_{x}\left(0\right)}$. The public key for access tree Γ is $gpk_{\Gamma }={\left\{D_{i},h_{i}\right\}}_{i\in \mathrm{leaves}(\Gamma )}$.

#### 5.2.4. *DelGen*

The original signer holding $sk_{\omega_{o}}$ selects $s_{o}\in Z_{q}$ and computes

$$\sigma_{o,0}=H_{2}{\left(w\right)}^{s_{o}}\cdot d_{o},\;\sigma_{o,0}^{\prime }=g^{s_{o}}.$$

Let $\omega^{*}$ denote the attribute set associated with leaves in Γ and $|\omega^{*}|=n$. For each attribute $i_{o}\in \omega^{*}$, it chooses $r_{i_{o}}^{\prime }\in Z_{q}$ and sets

$$\left(\sigma_{i_{o}0},\sigma_{i_{o}1}\right)=\left\{\begin{array}{cc} (d_{i_{o}0}\cdot H_{1}{\left(i_{o}\right)}^{r_{i_{o}}^{\prime }},\;d_{i_{o}1}\cdot g^{r_{i_{o}}^{\prime }}), & i_{o}\in \omega_{o}\cap \omega^{*},\\ (H_{1}{\left(i_{o}\right)}^{r_{i_{o}}^{\prime }},\;g^{r_{i_{o}}^{\prime }}), & i_{o}\in \omega^{*}/\omega_{o}\cap \omega^{*}. \end{array}\right.$$

The delegation is $\sigma_{o}=\left(\sigma_{o,0},{\left\{\sigma_{i_{o}0},\sigma_{i_{o}1}\right\}}_{i_{o}\in \Gamma },\sigma_{o,0}^{\prime }\right)$.

#### 5.2.5. *DelVer*

Given a warrant *w* and a delegation $\sigma_{o}$, the proxy signer recursively runs VerNode over the original signer’s leaf pairs to obtain $F_{o,root}$. The delegation is accepted if

$$e\left(g,\sigma_{o,0}\right)=F_{o,root}\cdot e\left(H_{2}\left(w\right),\sigma_{o,0}^{\prime }\right)\cdot Z,$$

and rejected otherwise.

The function *VerNode*$\left(\sigma_{o},gpk_{\Gamma },x\right)$ is a recursive procedure used to determine whether the original signer’s attributes satisfy the access tree Γ. It takes as input the delegation $\sigma_{o}$, the public key $gpk_{\Gamma }={\left\{D_{i},h_{i}\right\}}_{i\in \omega^{*}}$, and a node *x* in Γ, and outputs a group element $F_{o,root}\in G_{2}$ or ⊥.

If *x* is a leaf node, let $i_{o}=att\left(x\right)$ and compute $F_{x}=\frac{e(\sigma_{i_{o}0},D_{i_{o}})}{e(\sigma_{i_{o}1},h_{i_{o}})}$. If $F_{x}=1$, output ⊥; otherwise, output $F_{x}$.

If *x* is a non-leaf node, for each child node *z* of *x*, recursively compute $F_{z}=VerNode\left(\sigma_{o},gpk_{\Gamma },z\right)$. Let $S_{x}$ be any subset of child nodes such that $|S_{x}|=k_{x}$ and $F_{z}\neq ⊥$ for all $z\in S_{x}$. If no such subset exists, output ⊥. Otherwise, let $S_{x}^{\prime }=\left\{\mathrm{index}\left(z\right):z\in S_{x}\right\}$ and compute $F_{x}=\prod_{z\in S_{x}}F_{z}^{{∆}_{j,S_{x}^{\prime }}\left(0\right)}$, where j=index(z) and ${∆}_{j,S_{x}^{\prime }}\left(0\right)$ denotes the Lagrange interpolation coefficient at point 0. Finally, output $F_{x}$.

When the recursion reaches the root node, the algorithm outputs

$$F_{o,root}=e{\left(g,g_{2}\right)}^{r_{o}/\alpha \cdot p_{root}\left(0\right)}=e{\left(g,g_{2}\right)}^{r_{o}}.$$

#### 5.2.6. *Sign*

The proxy signer selects $s_{p}\in Z_{q}$ and computes

$$\sigma_{p,0}=\sigma_{o,0}\cdot H_{3}{\left(m\right)}^{s_{p}}\cdot d_{p},\;\sigma_{p,0}^{\prime }=g^{s_{p}}.$$

For each attribute $i_{p}\in \omega^{*}$, the signer chooses $r_{i_{p}}^{\prime \prime }\in Z_{q}$ and sets

$$\left(\sigma_{i_{p}0},\sigma_{i_{p}1}\right)=\left\{\begin{array}{cc} (d_{i_{p}0}\cdot H_{1}{\left(i_{p}\right)}^{r_{i_{p}}^{\prime \prime }},\;d_{i_{p}1}\cdot g^{r_{i_{p}}^{\prime \prime }}), & i_{p}\in \omega_{p}\cap \omega^{*},\\ (H_{1}{\left(i_{p}\right)}^{r_{i_{p}}^{\prime \prime }},\;g^{r_{i_{p}}^{\prime \prime }}), & i_{p}\in \omega^{*}/\omega_{p}\cap \omega^{*}. \end{array}\right.$$

The proxy signature $\sigma_{p}$ is $\sigma_{p}=\left(\sigma_{p,0},{\left\{\sigma_{i_{p}0},\sigma_{i_{p}1}\right\}}_{i_{p}\in \Gamma },\sigma_{p,0}^{\prime },{\left\{\sigma_{i_{o}0},\sigma_{i_{o}1}\right\}}_{i_{o}\in \Gamma },\sigma_{o,0}^{\prime }\right).$ The proxy signer sends ($m,w,\sigma_{p}$) to the verifier.

#### 5.2.7. *Verify*

The verifier runs VerNode on the original signer’s and proxy signer’s leaf pairs to obtain $F_{o,root}$ and $F_{p,root}$. The signature is accepted if

$$e\left(g,\sigma_{p,0}\right)=F_{p,root}\cdot e\left(H_{3}\left(m\right),\sigma_{p,0}^{\prime }\right)\cdot F_{o,root}\cdot e\left(H_{2}\left(w\right),\sigma_{o,0}^{\prime }\right)\cdot Z^{2},$$

and rejected otherwise.

## 6. EABPS Scheme Analysis

### 6.1. Correctness

We prove the correctness of our scheme in two steps. First, we verify that a delegation produced by *DelGen* is accepted by *DelVer*. From the function *VerNode*, we can compute $F_{o,root}=e\left(g,g_{2}^{r_{o}}\right)$. Then, we can proceed as follows:

$$\begin{array}{cc} e(g,\sigma_{o,0})= & e(g,H_{2}{\left(w\right)}^{s_{o}}g_{2}^{r_{o}+\alpha })\\ = & e\left(g,H_{2}{\left(w\right)}^{s_{o}}\right)\cdot e\left(g,g_{2}^{r_{o}}\right)\cdot e\left(g,g_{2}^{\alpha }\right)\\ = & F_{o,root}\cdot e\left(H_{2}\left(w\right),\sigma_{o,0}^{\prime }\right)\cdot Z \end{array}$$

Therefore a correctly formed delegation always passes *DelVer*, completing the correctness proof for the delegation phase.

Second, we prove that a proxy signature produced by *Sign* is accepted by *Verify*. From the function *VerNode*, we can also compute $F_{p,root}=e\left(g,g_{2}^{r_{p}}\right)$. Then, we can proceed as follows:

$$\begin{array}{cc} e(g,\sigma_{p,0})= & e(g,\sigma_{o,0}H_{3}{\left(m\right)}^{s_{p}}g_{2}^{r_{p}+\alpha })\\ = & e\left(g,\sigma_{o,0}\right)\cdot e\left(g,H_{3}{\left(m\right)}^{s_{p}}\right)\cdot e\left(g,g_{2}^{r_{p}}\right)\cdot e\left(g,g_{2}^{\alpha }\right)\\ = & F_{o,root}\cdot e\left(H_{2}\left(w\right),\sigma_{o,0}^{\prime }\right)\cdot Z\cdot F_{p,root}\cdot e\left(H_{3}\left(m\right),\sigma_{p,0}^{\prime }\right)\cdot Z\\ = & F_{p,root}\cdot e\left(H_{3}\left(m\right),\sigma_{p,0}^{\prime }\right)\cdot F_{o,root}\cdot e\left(H_{2}\left(w\right),\sigma_{o,0}^{\prime }\right)\cdot Z^{2} \end{array}$$

Hence, a proxy signature correctly formed as above will be accepted by *Verify*, completing the correctness proof for the proxy signing phase.

### 6.2. Security Analysis

#### 6.2.1. Unforgeability

In this subsection we give a formal proof that the proposed EABPS scheme has EUF–SP–CMA against $A_{\mathrm{II}}$ and $A_{\mathrm{III}}$ in the random oracle model.

**Theorem** **1.** *In the random oracle model, the proposed EABPS scheme has EUF-sP-CMA against $A_{\mathrm{II}}$, assuming the CDH assumption holds in group $G_{1}$.*

**Proof.**  It is assumed that there exists a PPT adversary $A_{\mathrm{II}}$ that breaks the EABPS scheme with non–negligible advantage ε. It is assumed that $A_{\mathrm{II}}$ makes $q_{H_{1}}$, $q_{H_{2}}$ and $q_{H_{3}}$ queries to the random oracles $O_{H_{1}}$, $O_{H_{2}}$, $O_{H_{3}}$, $q_{K}$ queries to $O_{KGPri}$, $q_{D}$ queries to $O_{DG}$ and $q_{S}$ queries to $O_{Sign}$. We use $A_{\mathrm{II}}$ to construct a PPT algorithm B that solves the CDH problem with non–negligible probability $\varepsilon^{\prime }$. Given a CDH instance $\left(g,X=g^{x},Y=g^{y}\right)$ in $G_{1}$, B computes $g^{xy}$ without knowing *x* or *y*. We denote $g_{1}=X=g^{x}$ and $g_{2}=Y=g^{y}$, and we let $Z=e\left(g_{1},g_{2}\right)=e{\left(g,g\right)}^{xy}$.

$A_{\mathrm{II}}$ declares the challenge access policy (attribute tree) $\Gamma^{*}$. Let $\omega^{*}$ denote the set of attributes labeling the leaves of $\Gamma^{*}$. $A_{\mathrm{II}}$ randomly chooses $\nu \in \{1,2,...,q_{H_{2}}\}$ and $\xi \in \{1,2,...,q_{H_{3}}\}$.

The adversary $A_{\mathrm{II}}$ queries some oracles. If the query value resides in the relevant list, the oracle returns the corresponding entry; otherwise, it generates the corresponding output and records both the query value and the output in the list. The random numbers $a_{i},b_{i},a_{i\prime }^{\prime },c_{i^{\prime }}^{\prime },a_{i^{\prime \prime }}^{\prime \prime },c_{i^{\prime \prime }}^{\prime \prime },r_{i}$ are chosen from $Z_{q}$.

$O_{H_{1}}$: $A_{\mathrm{II}}$ queries $O_{H_{1}}$ with the input *i*. B outputs $g^{b_{i}}$ if $i\in \omega^{*}$, and $g_{1}^{-b_{i}}g^{a_{i}}$ otherwise.

$O_{H_{2}}$: $A_{\mathrm{II}}$ queries $O_{H_{2}}$ with the input $w_{i^{\prime }}$. B outputs $g^{a_{i^{\prime }}^{\prime }}$ if $i^{\prime }=\nu$, and $g_{1}^{c_{i^{\prime }}^{\prime }}g^{a_{i^{\prime }}^{\prime }}$ otherwise.

$O_{H_{3}}$: $A_{\mathrm{II}}$ queries $O_{H_{3}}$ with the input $m_{i^{\prime \prime }}$. B outputs $g^{a_{i^{\prime \prime }}^{\prime \prime }}$ if $i^{\prime \prime }=\xi$, and $g_{1}^{c_{i^{\prime \prime }}^{\prime \prime }}g^{a_{i^{\prime \prime }}^{\prime \prime }}$ otherwise.

$O_{KGPri}$: $A_{\mathrm{II}}$ queries $O_{KGPri}$ with the attribute set ω which does not satisfy the challenge access structure, i.e., $\Gamma^{*}\left(\omega \right)\neq 1$.

For any validly queried ω, the set $\omega^{*}\cap \omega$ also fails to satisfy the access structure, which means $\Gamma^{*}\left(\omega^{*}\cap \omega \right)\neq 1$. It is assumed that *S* is the set of attributes corresponding to the leaf nodes in the access tree $\Gamma^{*}$. It satisfies the access structure $\Gamma^{*}$ and $\omega^{*}\cap \omega \subseteq S$. To construct the private key, B first selects $d=g_{2}^{r+x}$ uniformly at random.

For i∈S, the key components $d_{i0}$ and $d_{i1}$ are then simulated as $d_{i0}=g_{2}^{\frac{r}{x}}H_{1}{\left(i\right)}^{r_{i}}$ and $d_{i1}=g^{r_{i}}$.

For i∉S, B sets $r_{i}=\frac{{∆}_{0,S}\left(i\right)}{b_{i}}y+r_{i}^{\prime }$. The key components $d_{i0}$ and $d_{i1}$ are then simulated as $(d_{i0}=g_{2}^{\frac{{∆}_{0,S}\left(i\right)a_{i}}{b_{i}}+\frac{r}{x}}g^{-xy{∆}_{0,S}\left(i\right)}{\left(H_{1}\left(i\right)\right)}^{r_{i}^{\prime }}$ and $d_{i1}=g_{2}^{\frac{{∆}_{0,S}\left(i\right)}{b_{i}}}g^{r_{i}^{\prime }})$.

The core idea is that the private key for $\Gamma^{*}$ is generated by assigning a random polynomial p(·) to each node from the root downward. The root satisfies $p_{\mathrm{root}}\left(0\right)=x$, while for any other node, we set $p_{node}\left(0\right)=p_{\mathrm{parent}(node)}\left(\mathrm{index}\left(node\right)\right)$ and select the remaining coefficients randomly.

$O_{DG}$: To simulate $\left(g_{2}^{x}g_{2}^{r}H_{2}{\left(w\right)}^{s},{\left\{g_{2}^{\frac{r}{\alpha }}H_{1}{\left(i\right)}^{r_{i}},g_{1}^{r_{i}}\right\}}_{i\in \omega^{*}},g^{s}\right)$, B sets $s=\frac{-y}{c_{i}^{\prime }}+s^{\prime }$ where $s^{\prime }$ is a random value and $s^{\prime }\in Z_{q}$. It simulates $g_{2}^{\alpha }g_{2}^{r}H_{2}{\left(w\right)}^{s}=g_{2}^{r}{\left(g_{1}^{c_{i}^{\prime }}g^{a_{i}^{\prime }}\right)}^{s^{\prime }}g_{2}^{-\frac{a_{i}^{\prime }}{c_{i}^{\prime }}}$ and $g^{s}=g_{2}^{\frac{-1}{\alpha_{i}}}g^{s^{\prime }}$.

$O_{Sign}$: If $H_{2}\left(\omega_{i^{\prime }}\right)=g^{a_{i^{\prime }}^{\prime }}$ and $H_{3}\left(m_{i^{\prime \prime }}\right)=g^{a_{i^{\prime \prime }}^{\prime \prime }}$, B aborts. Otherwise, B simulates and outputs the signature.

Finally, $A_{\mathrm{II}}$ outputs a forged signature $\sigma_{p}^{*}$ on $m^{*}$ and $w^{*}$ with $\Gamma^{*}$. If $H_{2}\left(\omega^{*}\right)\neq g^{a_{\nu }^{\prime }}$ or $H_{3}\left(m^{*}\right)\neq g^{a_{\xi }^{\prime \prime }}$, B aborts. B constructs the solution to the CDH problem by processing the forged signature $\sigma_{p}^{*}$. This is achieved via a recursive algorithm that operates bottom-up on the challenge policy tree $\Gamma^{*}$.

Beginning with the leaf nodes, the algorithm leverages the structure of the forged signature components $\left\{\sigma_{i_{o}0}^{*},\sigma_{i_{o}1}^{*}\right\}$ and $\left\{\sigma_{i_{p}0}^{*},\sigma_{i_{p}1}^{*}\right\}$ to compute intermediate values. For each interior node, it then uses Lagrange interpolation to combine the values derived from its child nodes. This process continues until the root node is reached, at which point the term $R_{root}=g_{2}^{-r}$ is successfully isolated.

Finally, by combining this isolated term with the signature components $\sigma_{o,0}^{\prime *}$, $\sigma_{p,0}^{*}$ and $\sigma_{p,0}^{\prime *}$, the simulator is able to cancel all remaining unknown exponents. This allows for the direct computation of the CDH solution:

(1) $$g^{xy}=R_{root}\cdot {\left(\frac{\sigma_{p,0}^{*}}{{\left(\sigma_{p,0}^{\prime *}\right)}^{a_{\xi }^{\prime \prime }}{\left(\sigma_{o,0}^{\prime *}\right)}^{a_{\nu }^{\prime }}}\right)}^{\frac{1}{2}}$$

B solves the CDH problem with probability $\varepsilon^{\prime }\approx \frac{\varepsilon }{{\left(q_{H_{2}}\right)}^{2}q_{H_{3}}}$.Therefore, this EABPS scheme has EUF-SP-CMA against $A_{\mathrm{II}}$. □

**Theorem** **2.** *In the random oracle model, the proposed EABPS scheme has EUF-SP-CMA secure against $A_{\mathrm{III}}$, assuming the CDH assumption holds in group $G_{1}$.*

**Proof.**  The proof is similar to the proof Theorem 1. We compute $\varepsilon^{\prime }\approx \frac{\varepsilon }{q_{H_{2}}q_{H_{3}}}$. Therefore, this scheme has EUF-SP-CMA against $A_{\mathrm{III}}$. □

According to the Theorem 1 and Theorem 2, our EABPS scheme has EUF-SP-CMA.

#### 6.2.2. Attribute Privacy for Signer

**Theorem** **3.** *Our EABPS scheme achieves attribute privacy for original signers.*

**Proof.**  Let $\omega_{0}$ and $\omega_{1}$ be two distinct attribute sets such that $\Gamma \left(\omega_{0}\right)=\Gamma \left(\omega_{1}\right)=1$. We show that the delegations generated from these sets are computationally indistinguishable.

In the *DelGen* algorithm, the original signer introduces fresh randomness $s_{o}$ for $\left(\sigma_{o,0},\sigma_{o,0}^{\prime }\right)$ and independent random values $\left\{r_{i}^{\prime }\right\}$ for each leaf component $\left(\sigma_{i0},\sigma_{i1}\right)$. These randomizers computationally hide all key-related components contained in $\left(d_{i0},d_{i1}\right)$. Consequently, for every $i\in \omega^{*}$, the exposed leaf components $\left(\sigma_{i0},\sigma_{i1}\right)$ are distributed independently of whether the signer actually possesses attribute *i*, and are computationally indistinguishable from a fresh randomization of $\left(H_{1}{\left(i\right)}^{r_{i}^{\prime }},g^{r_{i}^{\prime }}\right)$.

Moreover, since both $\omega_{0}$ and $\omega_{1}$ satisfy the access structure Γ, the reconstruction performed in *VerNode* yields the same root value for both attribute sets. Thus the structure needed for verification leaks no distinguishing information.

Consequently, the complete delegation $\sigma_{o}=\left(\sigma_{o,0},{\left\{\sigma_{i0},\sigma_{i1}\right\}}_{i\in \omega^{*}},\sigma_{o,0}^{\prime }\right)$ is computationally indistinguishable under $\omega_{0}$ and $\omega_{1}$. The advantage ${\mathrm{Adv}}_{A}^{APOS}$ is negligible. Therefore, the scheme achieves attribute privacy for original signers. □

Using an argument analogous to that of Theorem 3, we obtain the following result.

**Theorem** **4.** *The EABPS scheme achieves attribute privacy for proxy signers.*

In summary, we can conclude that the EABPS scheme protects attribute privacy for signers.

#### 6.2.3. Collusion Resistance

It is important to note that our definition of unforgeability implies collusion resistance. In particular, no group of users or signers can combine their private keys or delegation information to generate a signature that none of them could produce individually. If a coalition of users were able to construct such an unauthorized signature, then one could build a new adversary that simulates the entire colluding group and outputs this signature as a valid forgery in the unforgeability game. This contradicts the security definition and therefore cannot occur.

Hence, any attempt by multiple users to pool their private keys or delegation does not provide them with additional signing capabilities. This establishes that the proposed EABPS scheme is inherently secure against collusion attacks.

### 6.3. Efficiency Analysis

In this subsection, we analyze feasibility of cryptographic constructions for UAV and assess the efficiency of our proposed EABPS scheme in comparison to related schemes: the ABPS-HL1 [29], ABPS-HL2 [30], and t-ABPS [31] schemes. First, we evaluate their computational costs by analyzing the execution times of the *Sign* and *Verify* algorithms. Second, we examine their communication overheads through a comparison of signature lengths.

#### 6.3.1. Feasibility of Cryptographic Constructions for UAV

We use hash operations, pairing operations, and the Java pairing-based cryptography (jPBC) library [33] to construct and implement our EABPS scheme. They are necessary for achieving the required security properties of ABPS schemes and suitable for the computational capabilities of UAV platforms.

(1) Hash operations

Hash functions are among the most lightweight cryptographic primitives supported by embedded processors. UAV platforms can compute them with low latency and energy consumption. In our scheme, hash functions play a fundamental role. These operations fully comply with the computational constraints of UAV nodes.

(2) Pairing operations

Pairing-based cryptography is necessary to enable expressive attribute-based delegation and proxy signing. In our scheme, UAVs are only required to execute the *Verify* algorithm that involves only a number of pairing operations. The feasibility of the pairing operation for UAV hardware is confirmed by our experimental data.

(3) jPBC

The jPBC library is adopted because it provides efficient and widely used implementations of bilinear pairings. By using jPBC on our platform, we obtain realistic estimates of pairing computational costs. Since UAVs only perform a number of pairing operations in the *Verify* algorithm, these experimental results demonstrate that the computational costs remain well within the capability range of typical UAV processors.

Therefore, hash operations, pairing operations, and the jPBC library employed in our EABPS scheme are not only essential from a security and functional perspective, but also fully compatible with the computational capabilities of UAVs.

#### 6.3.2. Computational Costs

The compared signature schemes employ fundamental cryptographic operations, including exponentiation, hashing, and bilinear pairing. We conduct a theoretical performance analysis to determine the number of these operations required for the *Sign* and *Verify* algorithms. The analytical results are summarized in Table 3 and Table 4, where *n* denotes the number of attributes and *k* indicates the threshold parameter.

For our EABPS scheme, the expressions in Table 3 and Table 4 are obtained directly from the step-based descriptions of the algorithms. Since each step explicitly specifies the cryptographic operations it invokes, we counted the exact occurrences of exponential operations, hash operations, and pairing operations. For example, in the *Sign* algorithm, the signer performs one hash operation and one exponential operation to compute $\sigma_{p,0}$, and one exponential operation to compute $\sigma_{p,0}^{\prime }$. It also performs 2n exponential operations and *n* hash operations to compute the components $\left(\sigma_{i_{p}0},\sigma_{i_{p}1}\right)$. Therefore, the signer performs a total of 2n+2 exponential operations and n+1 hash operations in the *Sign* algorithm. Using the same method, we derive the numbers of exponential operations, hash operations, and pairing operations in the *Verify* algorithm. For the schemes in [29,30,31], the expressions are derived in the same manner from the algorithmic descriptions provided in those constructions. These results form the expressions listed in Table 3 and Table 4.

**Figure 3:**
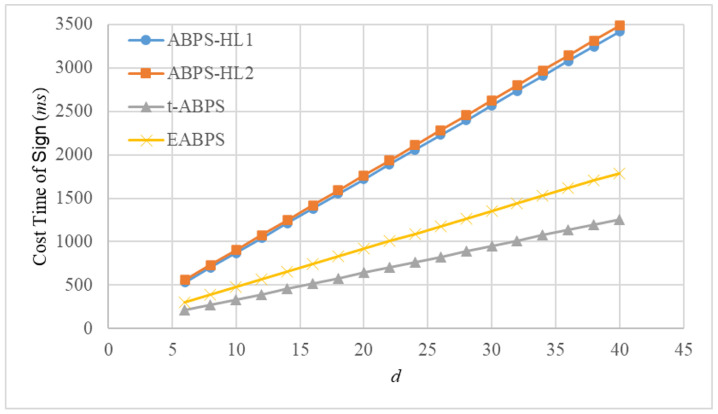
Computational time (ms) of algorithm *Sign* in different ABPS schemes.

**Figure 4:**
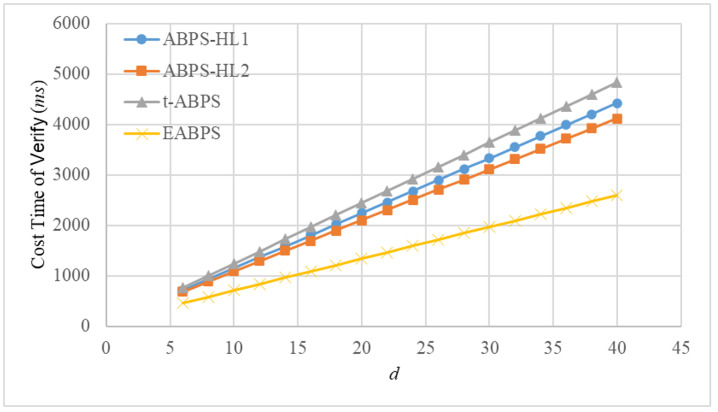
Computational time (ms) of algorithm *Verify* in different ABPS schemes.

The computational time of the fundamental operations was measured using the jPBC library. All experiments were conducted on a personal computer equipped with an Intel i5-13400 CPU and 16 GB of RAM. The implementation adopts a Type A pairing, where the parameter rBits is set to 160, representing the bit length of the group order, and qBits is set to 512, indicating the bit length of the underlying finite field. The experimental results demonstrate that the exponentiation, hash, and pairing operations require approximately 10.3 ms, 23.08 ms, and 10.59 ms, respectively. Based on both analytical evaluation and experimental measurements, we estimate the computational time of the *Sign* and *Verify* algorithms in these ABPS schemes. For ease of analysis and visualization, we assume n=d=2k. The corresponding results are presented in Figure 3 and Figure 4.

We provide the dependency formulas used to compute the execution time shown in Figure 3 and Figure 4. For each selected value of *d*, based on the assumption n=d=2k, we obtain the corresponding number of exponential operations $N_{exp}\left(d\right)$, hash operations $N_{hash}\left(d\right)$, and pairing operations $N_{pair}\left(d\right)$ required by the *Sign* algorithm. Therefore, the total running time for *Sign* at parameter *d* is computed as $T_{Sign}\left(d\right)=10.3N_{exp}\left(d\right)+23.08N_{hash}\left(d\right)+10.59N_{pair}\left(d\right)$. The same dependency expression is used for *Verify*.

Because each operation count in Table 3 grows linearly with *d*, the resulting running time $T_{Sign}\left(d\right)$ and $T_{Verify}\left(d\right)$ also grow linearly. Thus, the linearity observed in Figure 3 and Figure 4 reflects the property of the underlying construction.

Figure 3 illustrates the computational time of the *Sign* algorithm for four ABPS schemes, namely ABPS-HL1, ABPS-HL2, t-ABPS, and the proposed EABPS, as the number of attributes *d* increases. All schemes exhibit a roughly linear growth in signing cost, which is consistent with the increasing number of operations required for larger attribute sets. However, significant differences appear in their overall efficiency. ABPS-HL2 incurs the highest computational overhead, while ABPS-HL1 performs moderately better but still suffers from substantial computational burden. In comparison, EABPS consistently achieves a lower signing cost than both ABPS-HL1 and ABPS-HL2 for all examined values of *d*. This performance improvement stems from the optimized signing procedure in the EABPS, which reduces redundant exponential operations. Although t-ABPS demonstrates the lowest cost among all schemes, its performance advantage comes at the expense of limited expressiveness and reduced support for flexible attribute policies. Overall, the results confirm that EABPS provides a favorable balance between expressiveness and efficiency, making it a more practical solution for real-world scenarios that require secure and fine-grained proxy signing capabilities.

Figure 4 shows the verification time of four ABPS schemes as the number of attributes *d* increases. All schemes exhibit an approximately linear growth pattern, consistent with the increasing number of operations required during verification. However, the efficiency differences among the schemes are substantial. The ABPS-HL1, ABPS-HL2, and t-ABPS schemes incur relatively high verification overhead, with t-ABPS being slightly more expensive.

In contrast, the proposed EABPS scheme consistently achieves the lowest verification cost across all tested values of *d*. This efficiency gain results from the optimized verification design in EABPS, which reduces redundant hash operations. As a result, the EABPS scheme provides a more practical balance between verification efficiency and policy expressiveness. These results confirm that EABPS significantly enhances verification performance while maintaining flexible and secure attribute-based proxy signing capabilities. Moreover, these results make EABPS particularly suitable for deployment in real-world scenarios where a large number of verifications must be performed efficiently, such as UAV networks and distributed authentication systems.

**Figure 5:**
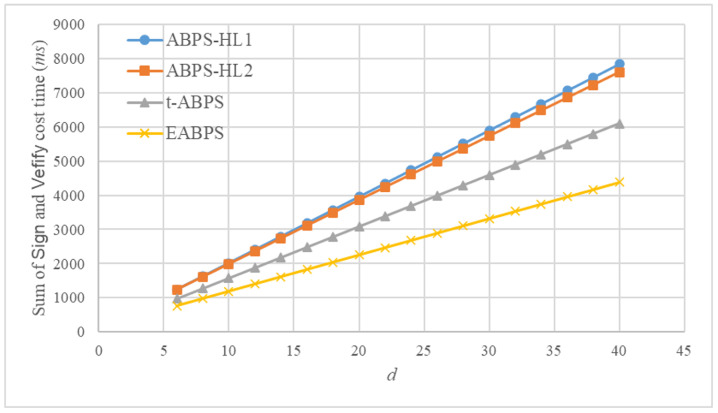
Total computational time (ms) of the algorithms *Sign* and *Verify* in different ABPS schemes.

Figure 5 presents the combined computational time of the *Sign* and *Verify* algorithms for four ABPS schemes as the number of attributes *d* increases. All schemes exhibit a roughly linear increase in total computation cost. Among the compared schemes, ABPS-HL1 and ABPS-HL2 incur the higher overall cost, with their execution time rising rapidly as *d* grows. Although t-ABPS performs more efficiently than ABPS-HL1 and ABPS-HL2 schemes, its comparatively lower total cost stems from its restricted expressiveness and inability to support rich attribute policies.

In contrast, the proposed EABPS scheme consistently achieves the lowest total computational time across all tested values of *d*. The improvement mainly results from the optimized design of its verification procedure. As a consequence, the EABPS scheme delivers a substantially more efficient end-to-end signing and verification process while preserving full attribute expressiveness and strong security guarantees. These findings confirm that the EABPS scheme offers a superior balance between computational efficiency and functional capability, making it well suited for practical deployment in large-scale, attribute-driven application environments, such as UAV networks and distributed authentication systems.

In addition, it is essential to discuss the applicability of our experimental results to UAV hardware platforms. Although our implementation and measurements are conducted on a desktop computer, recent studies show that many modern UAVs are equipped with embedded processors. The computational complexity of our scheme depends primarily on exponential operations, hash operations, and pairing operations. The relative efficiency and scalability trends observed in our experiments remain valid for UAV platforms, even though the absolute running time would scale according to the processor performance. Hence, the proposed EABPS scheme is suitable for deployment on typical UAV hardware.

#### 6.3.3. Communication Costs

Since a signer must transmit a digital signature to the verifier, the signature size is regarded as the communication cost. A theoretical analysis of the signature length for these schemes is presented in Table 5.

**Figure 6:**
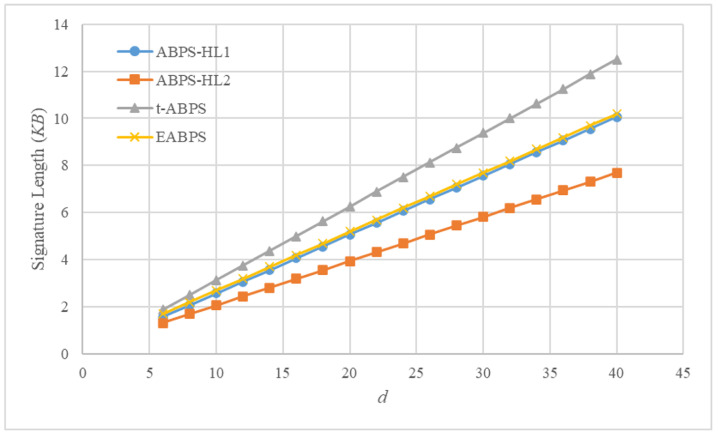
Comparison of signature length of the schemes (*KB*).

We assume n=d=2k and |G|=512 bits. The results are illustrated in Figure 6.

Figure 6 compares the signature lengths of four ABPS schemes as the number of attributes *d* increases. All schemes exhibit a linear growth pattern, reflecting the fact that signatures contain attribute-related components whose number scales with the size of the underlying attribute set. However, notable differences exist in the communication costs. The t-ABPS scheme yields the largest signature size across all tested values of *d*. ABPS-HL1 and ABPS-HL2 generate smaller signatures.

In contrast, the proposed EABPS scheme yields signature lengths larger than those of ABPS-HL1 and ABPS-HL2 but smaller than t-ABPS. This intermediate position results from design trade-offs in the EABPS scheme. These trade-offs arise from the structural differences between our expressive tree-based access policies and the simpler threshold-based designs used in related ABPS schemes. Expressive policies inherently require additional structural overhead, which modestly increases the overall signature size. Nevertheless, the proposed EABPS scheme is suitable for UAV environments, where fine-grained authorization and authentication are essential, and where slightly larger signature sizes are acceptable in exchange for enhanced functionality.

## 7. Conclusions

We presented an EABPS scheme designed to address the security and privacy challenges inherent in UAV networks. Our scheme enables fine-grained authorization and authentication through flexible attribute policies, ensuring that only entities whose attributes satisfy a predefined access structure are permitted to generate valid proxy signatures. The EABPS scheme further preserves the attribute privacy for both original and proxy signers, effectively decoupling signatures from explicit identities and mitigating privacy risks.

We conducted security analysis to demonstrate that the proposed scheme achieves unforgeability and attribute privacy. Experimental evaluation confirmed that the EABPS scheme offers improved efficiency compared with existing ABPS constructions while maintaining strong expressiveness and lower communication overhead. These results indicate that the EABPS scheme provides a practical cryptographic construction for secure delegation, message authentication, and privacy-preserving communication in UAV networks.

Future work will explore optimizing the scheme for large-scale, heterogeneous UAV environments, integrating lightweight cryptographic techniques for resource-constrained UAVs, and extending the construction to support revocation and dynamic attribute updates.

## Figures and Tables

**Table 1:** Comparison of representative security solutions.

Security Solutions	Integrity	Authentication	Protection of Signer’s Identity
Din et al. [5]	√	√	×
Qu et al. [10]	√	√	×
Khan et al. [16]	√	√	×

**Table 2:** Comparison of representative ABPS schemes.

Security Solutions	Integrity	Authentication	Protection of Signer’s Identity	Access Structure
ABPS-HL1 [29]	√	√	√	Threshold
ABPS-HL2 [30]	√	√	√	Threshold
t-ABPS [31]	√	√	√	Threshold

**Table 3:** Amount of some operations in *Sign*.

Signature Schemes	Exponential Operation	Hash Operation	Pairing Operation
EABPS	2n+2	n+1	0
t-ABPS	3n	1	0
ABPS-HL1	6d	d+1	0
ABPS-HL2	2n+4d − 2k+2	n+d − k+1	0

**Table 4:** Amount of some operations in *Verify*.

Signature Schemes	Exponential Operation	Hash Operation	Pairing Operation
EABPS	2n	2	4n+3
t-ABPS	2d	2d+2	5d
ABPS-HL1	2d	2d+2	4d+1
ABPS-HL2	0	2(n+d − k+1)	2(n+d − k)+3

**Table 5:** Signature length of different ABPS schemes.

Signature Schemes	Signature Length
EABPS	(4n+3)|G|
t-ABPS	5n|G|
ABPS-HL1	(4d+1)|G|
ABPS-HL2	(2(n+d−k)+3)|G|

## Data Availability

Dataset available on request from the authors.
